# Simultaneous detection of *Plasmodium vivax dhfr*, *dhps*, *mdr1* and *crt*-*o* resistance-associated mutations in the Colombian Amazonian region

**DOI:** 10.1186/s12936-018-2286-5

**Published:** 2018-03-27

**Authors:** Juan Ricardo Cubides, Paola Andrea Camargo-Ayala, Carlos Hernando Niño, Diego Garzón-Ospina, Anggie Ortega-Ortegón, Estefany Ospina-Cantillo, María Fernanda Orduz-Durán, Manuel Elkin Patarroyo, Manuel Alfonso Patarroyo

**Affiliations:** 1 Molecular Biology and Immunology Department, Fundación Instituto de Inmunología de Colombia (FIDIC), Cra. 50 # 26-20, Bogotá, Colombia; 2 School of Medicine and Health Sciences, Universidad del Rosario, Carrera 24#63C-69 Bogotá, Colombia; 3 School of Medicine, Universidad Nacional de Colombia, Avenida Carrera 30 # 45, Bogotá, Colombia

**Keywords:** Malaria, *Plasmodium vivax*, Antimalarial drug resistance, Amazonian region, Colombia, *pvdhfr*, *pvdhps*, *pvmdr1* and *pvcrt*-*o*

## Abstract

**Background:**

Malaria continues being a public health problem worldwide. *Plasmodium vivax* is the species causing the largest number of cases of malaria in Asia and South America. Due to the lack of a completely effective anti-malarial vaccine, controlling this disease has been based on transmission vector management, rapid diagnosis and suitable treatment. However, parasite resistance to anti-malarial drugs has become a major yet-to-be-overcome challenge. This study was thus aimed at determining *pvmdr1*, *pvdhfr*, *pvdhps* and *pvcrt*-*o* gene mutations and haplotypes from field samples obtained from an endemic area in the Colombian Amazonian region.

**Methods:**

Fifty samples of parasite DNA infected by a single *P. vivax* strain from symptomatic patients from the Amazonas department in Colombia were analysed by PCR and the *pvdhfr*, *pvdhps*, *pvmdr1* and *pvcrt*-*o* genes were sequenced. Diversity estimators were calculated from the sequences and the haplotypes circulating in the Colombian Amazonian region were obtained.

**Conclusion:**

*pvdhfr*, *pvdhps*, *pvmdr1* and *pvcrt*-*o* genes in the Colombian Amazonian region are characterized by low genetic diversity. Some resistance-associated mutations were found circulating in this population. New variants are also being reported. A selective sweep signal was located in *pvdhfr* and *pvmdr1* genes, suggesting that these mutations (or some of them) could be providing an adaptive advantage.

## Background

Malaria continues being a worldwide problem; it is a vector-born disease (VBD) transmitted by parasites from the genus *Plasmodium* [1]. About 40% of the world’s population (around three billion people) are currently at risk of suffering from this disease. Malaria causes more than 200 million clinical cases and about 400,000 deaths each year [1]. The climatic and geographical conditions in Colombia enable the transmission of this infection [2], and both *Plasmodium vivax* and *Plasmodium falciparum* are prevalent.

There are three large *Plasmodium* endemic foci in Colombia: Urabá–lower Cauca–southern Córdoba, the Pacific coast and the Orinoquía–Amazonía transition region; such regions favour malarial incidence and dispersion due to their environmental conditions and social, political and cultural variables [1, 3]. The main difficulty in controlling *P. vivax* lies in the need for treating both blood stage and latent liver forms (hypnozoites), the latter causing relapses months or years after initial infection [4].

First-line treatment for uncomplicated *P. vivax* malaria in Colombia is currently based on a combination of chloroquine (CQ) for eliminating blood stages (schizonts) and primaquine (PQ) for liver stages (hypnozoites) [5]. In other countries, treatment is also based on the use of sulfadoxine–pyrimethamine (SP) [6]. Although these drugs remain effective overall, cases of resistance have been recorded during the last few years, making this one of the greatest problems in controlling and eliminating malaria. The first report of CQ-resistant *P. vivax* strains in Latin-America was published in 1989 (including Colombia and Brazil) [7]; however, resistance targets have not been clearly established. Regarding *P. falciparum*, parasite genes associated with resistance to anti-malarial drugs have been established with greater certainty. Simple, double or quadruple mutations in different genes enable the parasite to cope with anti-malarial drugs. Mutations in *pfcrt*, *pfmdr1*, *pfdhfr* and *pfdhps* genes have arisen/been fixed in several parasite populations and, since they confer drug resistance, this facilitates their dispersion.

There may be a clear association between *pfcrt*, *pfmdr1*, *pfdhfr* and *pfdhps* allele variants and drug resistance, but such association is not clear regarding *P. vivax*. Nevertheless, genetic studies of *P. vivax* drug susceptibility in different parts of the world [8–13] have identified point mutations in orthologous genes (*pvcrt*-*o*, *pvmdr1*, *pvdhfr* and *pvdhps*) to those associated with resistance in *P. falciparum*, providing potential markers of resistance to *P. vivax* anti-malarial drugs [14–16]. Molecular biology techniques provide a valid alternative for identifying resistance-associated mutations regarding anti-malarial drug therapy in different populations and could therefore be used as a tool for surveillance of *P. vivax* drug resistance.

Very few studies have been made in Colombia regarding the search for polymorphisms in these genes. A 2015 evaluation in Colombia’s Córdoba department [17] stated that the *P. vivax* population was more genetically diverse than it had been thought. However, some genome regions had low diversity due to a selective sweep associated with the PvDHPS A383G allele [17] which, in turn, has been associated with sulfadoxine resistance [18]. The region surrounding PvDHFR did not have a loss of genetic diversity, as with PvDHPS; nevertheless, two alleles (S58R and S117N) [17] were reported to be associated with SP resistance [11]. Five non-synonymous mutations have been reported in *pvdmr1*; the mutation triggering change Y976F has been associated with reduced sensitivity to CQ [19]. However, other studies have not found susceptibility to CQ, despite Y976F being present. This phenotype has been reported in the north of Colombia [17], although such marker’s role in resistance has not been completely clarified [16]. The Colombian Amazon region is another focus for malaria as the people inhabiting the area live far away from city centres (i.e. lacking basic services and sanitation), their insecticide use is infrequent and access to healthcare centres is limited, making them more vulnerable to acquiring the disease and putting them at greater risk of not receiving timely treatment. Bewilderingly, there have been no studies regarding the presence of genotypes associated with resistance to the anti-malarial drugs used against *P. vivax* in this region. This study was thus aimed at evaluating the genetic diversity of *pvmdr1*, *pvdhfr*, *pvdhps* and *pvcrt*-*o* fragments in *P. vivax* parasites from Colombia’s Amazonian region, thereby providing significant information for the molecular surveillance of drug-resistant *P. vivax*.

## Methods

### Study areas

The samples used in this study were taken from several settlements in the municipalities of Leticia and Puerto Nariño in Colombia’s Amazonas department after volunteers had signed informed consent forms. These municipalities are located along the Banks of the Amazonas and Loretoyacu rivers on the Amazonian frontier with Peru and Brazil. All sample taking-related procedures and managing patients were approved by the Universidad del Rosario’s School of Medicine and Health Sciences’ Research Ethics Committee (resolution CEI-ABN026-000161).

### Extracting parasite DNA and confirming single *Plasmodium vivax* infection

Genomic DNA samples were extracted from FTA card-stored blood of *Plasmodium* spp. infected patients using a Pure Link Genomic DNA mini kit (Invitrogen), following the manufacturer’s specifications. The samples were eluted in 50 µL final volume of buffer containing 10 mM Tris–HCl, at pH 9.0 and 0.1 mM EDTA. *P. vivax* infection was confirmed, as described previously [20].

Samples positive for *P. vivax* were explored for selecting those which were only infected by a single strain. A *pvmsp3α* fragment was amplified and sequenced, as previously described [21, 22]. The resulting electropherograms were assembled and revised using CLC Genomics Workbench (v.3) [23]. Samples having overlapping peaks in electropherograms were discarded; only samples whose electropherograms showed single peaks were involved in analysis.

### Amplifying, purifying and sequencing *pvmdr1*, *pvdhfr*, *pvdhps* and *pvcrt*-*o*

Fragments from each gene associated with resistance were amplified using 50 samples confirmed as being infected by just one *P. vivax* strain, using previously described primers (Table 1) and thermal profiles *pvmdr1* [19], *p*v*dhfr* [24], *pvdhps* [25] and *pvcrt*-*o* [26]. PCR products were then purified using a Wizard SV Gel and PCR Clean-up System kit (Promega), following the manufacturer’s indications. Purified products were then bidirectionally sequenced using the ABI 3730 XL system (Macrogen, Seoul, South Korea).

**Table 1:** Primers used for sequencing *msp3*-*α*, *pvmdr1*, *pvdhfr*, *pvdhps* and *pvcrt*-o

Gene of interest	Primers used for sequencing		Size (bp)
	Primer	Sequence	
*msp3*-*α:* [21]	P1	5′-CAGCAGACACCATTTAAGG-3′	2194
	P2	5′-CCGTTTGTTGATTAGTTGC-3′	
	N1	5′-GACCAGTGTCATACCATTAACC-3′	1895
	N2	5′-ATACTGGTTCTTCGTCTTCAGG-3′	
*pvcrt*-*o:* [26]	F	5′-AAGAGCCGTCTAGCCATCC-3′	1186
	R	5′-AGTTTCCCTCTACACCCG-3′	
*pvdhps*: [25]	*Pv*DHPS-D	5′-GGTTTATTTGTCGATCCTGTG-3′	1301
	*Pv*DHPS-B	5′-GAGATTACCCTAAGGTTGATGTATC-3′	
*pvmdr1*: [19]	14-F	5′-CCCTCTACATCTTAGTCATCG-3′	932
	14-R	5′-TGGTCTGGACAAGTATCTAAAA	
	976-F	5′-GGATAGTCATGCCCCAGGATTG-3′	604
	976-R	5′-CATCAACTTCCCGGCGTAGC-3′	
*pvdhfr*: [24]	PvDA	5′-ACCGCACCAGTTGATTCCTAC-3′	1018
	PvDB	5′-TGTTAAAGCTGAAGTACACGAG-3′	
	PvDF	5′-ATGGAGGACCTTTCAGATGT-3′	785
	PvDR	5′-AACGCATTGCAGTTCTCCGA-3′	

### Analysing *pvmdr1*, *pvdhfr*, *pvdhps* and *pvcrt* sequences

The resulting electropherograms were corrected and assembled using CLC Genomics Workbench (v.3) [23] for obtaining a consensus sequence per sample and per gene. Consensus sequences were compared to the respective Salvador I (Sal-I) strain reference sequences having an antimalarial drug sensitivity profile (PlasmoDB access numbers: PVX_080100 for *pvmdr1*, PVX_089950 for *pvdhfr*, PVX_123230 for *pvdhps* and PVX_087980.1 for *pvcrt*-*o*). The respective amino acid (aa) sequence was inferred for each nucleotide sequence obtained from the Colombian Amazonian region, as well as Sal-I strain sequences and their orthologues in *Plasmodium cynomolgi* (a species phylogenetically-related to *P. vivax*; PlasmoDB access numbers: PCYB_101870 for *pvmdr1*, PCYB_053150 for *pvdhfr*, PCYB_143740 for *pvdhps* and PCYB_011610-t26_1 for *pvcrt*). The Muscle algorithm was then used for aligning them; this aa alignment was then taken as reference for inferring nucleotide alignment, using *PAL2NAL* software [27].

### Genetic diversity and the effect of natural selection

DnaSP software (v.5.10.00) was used for calculating several genetic diversity estimators from *pvmdr1*, *pvdhfr*, *pvdhps* and *pvcrt* nucleotide alignment, such as the segregating sites number, the haplotypes number, nucleotide and haplotype diversity and nucleotide polymorphism [28]. The modified Nei–Gojobori method [29] was used with MEGA software (v.7) [30] for evaluating selective pressure by calculating synonymous (d_S_) and non-synonymous (d_N_) substitution rates. Tajima [31], Fu and Li [32], and Fay and Wu estimators [33] were used for evaluating deviations from the neutral model of molecular evolution.

## Results

### *pvmdr1*, *pvdhfr*, *pvdhps* and *pvcrt-o* polymorphism

**Fig. 1:**
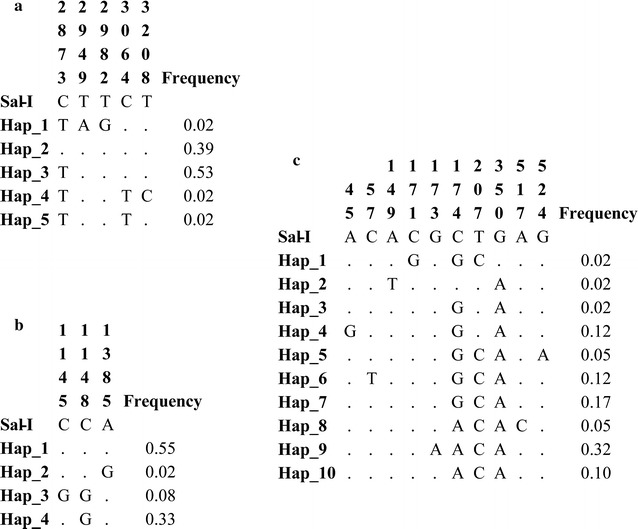
DNA haplotypes found in the Colombian Amazon region. **a** Aligning *pvmdr1* gene non-conserved residues. **b** Aligning *pvdhps* non-conserved residues. **c** Aligning *pvdhfr* non-conserved residues. Dots indicate conserved residues. Nucleotide positions are indicated according to the *P. vivax* Sal-I strain numbering (PlasmoDB accession PVX_089950 for *pvmdr1*, PVX_123230 for *pvdhps* and PVX_080100 for *pvdhfr*)

**Fig. 2:**
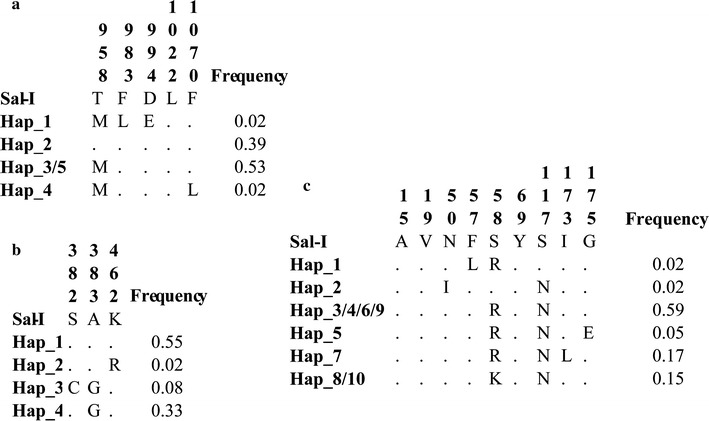
Amino acid haplotypes found in the Colombian Amazon region. **a** PvMDR1 alignment. **b** PvDHPS alignment. **c** PvDHFR alignment. Dots indicate conserved residues. Amino acid positions are indicated according to *P. vivax* Sal-I strain numbering (PlasmoDB accession PVX_089950 for PvMDR1, PVX_123230 for PvDHPS and PVX_080100 for PvDHFR)

Fifty samples infected by a single population of *P. vivax* were used for evaluating the polymorphism of several fragments from genes associated with resistance. Five polymorphisms were observed for *pvdmr1* (Fig. 1), four being non-synonymous and just one being synonymous; this gene’s nucleotide diversity (π) was 0.0013 and no substitution producing a change in aa Y976F (one of the first chemoresistance molecular marker candidates) [8] was found in samples from the Colombian Amazonian region. However, 62% of samples evaluated had T958M polymorphism and variant D994E was observed in 54% of the samples (Table 2 and Fig. 2). F983L and L1022 polymorphisms, encoding singleton sites, were observed as well as phenylalanine being replaced by leucine in position 1070 (Table 2 and Fig. 2). Concerning DNA, the 5 polymorphisms produced 5 different haplotypes (Fig. 1), haplotype 3 occurring most frequently (53%), followed by haplotype 2 (39%). The synonymous substitution of a cytosine for a thymine was found in position 3064, located in position 1 of the codon triplet (2% frequency).

**Table 2:** *P. vivax* resistance-associated molecular marker prevalence

Codon	% of mutations	Mutation	Protein change
*mdr1*			
2873	62	ACG>ATG	T958M
2949	38	TTT>TTA	F983L
2982	54	TTC>CTC	D994E
3064	2	CTA>TTA	L1022
3208	2	TTC>CTC	F1070L
*dhfr*			
45	12.2	GCA>GCG	A15
57	12.2	GTC>GTT	V19
149	2.44	AAC>ATC	N50I
171	2.44	TTC>TTG	F57L
173	31.7	AGC>AAG	S58K
174	97.5	AGC>AGG/AGA	S58R
207	82.9	TAT>TAC	Y69
350	97.6	AGC>AAC	S117N
517	4.88	ATT>CTT	I173L
524	4.88	GGA>GAA	G175E
*dhps*			
1145	8.3	TCC>TGC	S382C
1148	41.6	GCC>GGC	A383G
1385	2	AAG>AGAG	K462R

*pvdhfr* fragments could only be obtained in 41 of the 50 samples; 10 polymorphisms were observed (Fig. 1), with a π = 0.0037 and 10 different haplotypes (Fig. 1). Some polymorphisms observed coincided with those reported in other populations; 97.6% of the samples evaluated here had S117N substitutions, Y69 had 82.9% prevalence and S58K 31.7%, whilst the S58R polymorphism occurred in 97.5% of the samples (Table 2 and Fig. 2). Regarding DNA haplotypes, haplotype 9 occurred with the greatest frequency (32%) compared to haplotypes 1, 2 and 3 (Fig. 1). Synonymous substitutions were observed in changes A15 and V19 (12.2%).

A *pvdhps* fragment was obtained in 48 of the 50 samples; three polymorphisms and 4 haplotypes were observed in this gene (Fig. 1), the π was 0.0011, all polymorphisms being non-synonymous. The A383G substitution occurred in 41.6% of samples, whilst polymorphism A382C was only observed in 8.3% of them (Table 2 and Fig. 2). Haplotype 1 occurred in 55% of samples compared to haplotype 4 in 33% of them (Fig. 1). Finally, there was no polymorphism in *pvcrt*-*o*, showing that all isolates from the Colombian Amazonian region analysed here had 100% identity with the Sal-1 reference strain.

### Natural selection in genes associated with resistance

Several tests based on the neutral molecular evolution model were performed to determine whether fragments from genes associated with resistance were under some selective pressure; d_N_–d_S_ ratio values had no significant values (Table 3). Tajima, and Fu and Li tests revealed gave negative values; however, they were not significant. Nevertheless, Fay and Wu H estimator gave significant *P. vivax pvdhfr* and *pvdmr1* negative values for the population in the south of the Colombian Amazonian region (Table 3).

**Table 3:** Genetic diversity estimators and neutral tests

n	Gene	Sites	Ss	H	Hd	θ	π	d_N_–d_S_	Fay and Wu
									H
41	*dhfr*	558	10	10	0.84	0.0040	0.0037	− 0.006*	− 3.64^†^
48	*dhps*	648	3	4	0.56	0.0010	0.0011	0.002	0.32
50	*mdr1*	522	5	5	0.55	0.0021	0.0013	0.001	− 1.50^†^
33	*crt*-*o*	375	0	0	0.00	0.0000	0.0000	–	–

*n* total sequences used for analysis, *Sites* total sites used to rule out gap sites, *Ss* amount of segregating sites, *H* amount of haplotypes, *Hd* haplotype diversity, *θ* nucleotide polymorphism per site, *π* nucleotide diversity per site, *d*_*N*_–*d*_*S*_ ratio of non-synonymous to synonymous nucleotide polymorphism

* *p* = 0.038

^†^*p* < 0.01

## Discussion

*Plasmodium vivax* genetic diversity is an important challenge which must be overcome in attempts to control this infection [34]. Such diversity could enable the parasite to evade host immune responses and confer an adaptive advantage for resisting anti-malarial drugs [35].

The transmission of *Plasmodium* species in Colombia varies according to each geographical area, enabling several of them to cohabit in the same area. *Plasmodium falciparum/P. vivax* multiple infections could go unnoticed during diagnosis [6, 20] and thus the attending doctor cannot prescribe suitable treatment for patients; in turn, this could exercise selective pressure on parasite populations. Choosing the right treatment is thus crucial for preventing the appearance and propagation of resistance [1]. The samples analysed here came from a tri-border region involving Colombia, Peru and Brazil. First-line treatment for uncomplicated *P. vivax* infections is similar for the three countries, where a combination of CQ and PQ is used [5, 36, 37]. Previous studies have reported polymorphisms associated with resistance to anti-malarial drugs in South and Central America [7, 10].

Polymorphisms have been found in Central America in *pvmdr1* and *pvdhfr* which may be related to a loss of *P. vivax* susceptibility to anti-malarial drugs [10]. Nevertheless, no in vitro susceptibility studies have been reported so far to support such association. Cases of CQ therapeutic failure concerning treating *P. vivax* malaria have been documented in South America. Around 12% of therapeutic failure due to CQ-resistant parasites and 6.4% due to mefloquine-resistant parasites have been reported in Brazil’s Amazon region [38]. Taking into account that the sampling site in this study was near the tri-border area, the interchange of parasite strains carrying resistance mutations was likely and such haplotypes would become dispersed throughout neighbouring endemic areas. Although, *P. vivax* CQ resistance mechanisms have not been fully elucidated, they could involve various genetic loci. *Plasmodium falciparum pfcrt*-*o* and *pfmdr1* variants have been identified as being associated with CQ resistance; these genes’ orthologues in *P. vivax* have thus been evaluated for determining whether they might be associated with resistance [39].

Previous studies have found that PvMDR1 Y976F and F1076L variants seem to have been associated with CQ resistance [40]. One such polymorphism (variant Y976F) has been found to occur frequently in the east of the Brazilian Amazonian region [7]. Nevertheless, the role of Y976F in CQ resistance is not yet clear [16]. Neither Y976F nor F1076L have been observed in the Colombian Amazonian region’s population. Even so, other PvMDR1 polymorphisms were found in this population which have not been reported previously and could thus be substitutions in the strains circulating in the Colombian Amazon region. This suggested that haplotypes segregating in the Brazilian Amazonian region are different to those segregating in the Colombian equivalent and, therefore, gene flow between the Brazilian and Colombian Amazon regions seems to be limited or restricted. Further analysis is required to corroborate whether additional substitutions found in Colombia are related to clinical-resistance phenotypes.

Various mutations in *pvdhfr* and *pvdhps* have been associated with resistance to SP [41]. SP in Colombia and Brazil is not currently used as first-line treatment for *P. vivax* infection, even though having been included in Colombian Ministry of Health guidelines towards the end of the 1990s [5]. However, SP is used as the drug of choice for treating *P. falciparum* in Colombia [37, 42] and in northern Perú [43]. Diagnosis in the three countries is by thick smear; however, several reports have highlighted under-recording of cases by this method and a high rate of coinfection on the Colombia–Brazil–Perú frontier [20, 44]. This diagnostic technique’s inherent limitations and the large amount of misdiagnosed co-infections could affect selecting the proper treatment, thereby creating selective pressure on the parasite, leading to the fixing of SP resistance in *P. vivax* strains [45], even if SP may not be being used for treating vivax malaria. Polymorphisms found in PvDHFR (F57L/I, S58R, T61M and S117T/N) usually appear in patients having therapeutic failure [46]. Parasites having double substitutions (F57L–S117N), triple substitutions (5F7L–5S8R–S117N) and quadruple substitutions (F57L–S58R–T61M–S117T) have thus been associated with SP resistance [10, 47, 48]. Ten different polymorphisms were found in the Amazon region evaluated in this work; double substitutions (S58R–S117N) were observed in 59% of them. It has been proposed that the current use of SP in Colombia has exercised selective pressure on both *P. falciparum* and *P. vivax* [49]; validation follow-up is thus recommended to observe its efficacy as treatment. A triple mutation haplotype was observed (S58R–S117N–I173L) in 17% of samples; however, it is not clear whether this haplotype could be associated with resistance.

PvDHPS polymorphisms in aa 382, 383, 512, 553 and 585 (homologues for *P. falciparum* positions 436, 437, 540, 581 and 613, respectively) [26] seem to be involved in resistance to sulfadoxine. A high S382C mutation prevalence in chloroquine and mefloquine-resistant parasites has been observed in Brazil’s Amazon region [38, 50]. A383G substitution prevalence was 41.6%, the most prevalent in this study, unlike S382C and K462R mutations; this could have been related to changes in aa position 382 and 383 which are frequent in regions having high SP use [51]. In addition to the aforementioned polymorphisms, new *pvdhfr* and *pvdhps* variants and haplotypes were found in *P. vivax* strains from the Colombian Amazonian region.

*pvcrt* is an orthologue of *pfcrt* whose association with resistance to CQ is controversial [52]; CRT has shown no contribution whatsoever to resistance to chloroquine regarding *P. vivax*, even though sensitive or resistant *P. vivax* parasites cause substitution 76K [53]. Concerning the Colombian Amazonian region this gene fragment was seen to be highly conserved, having 100% identity with the *P. vivax* Sal-1 strain which is susceptible to anti-malarial drugs.

### Selective sweep could be taking place, fixing advantageous variants

Point mutations or determined haplotypes in *P. falciparum* that alter *dhfr*, *dhps*, *mdr1* and *crt*-*o* protein products, enable the parasite to resist several drugs. Such mutations are usually fixed rapidly in populations due to a selective advantage causing DNA regions containing such genes to have low genetic diversity (such effect is known as selective sweep). The *pvdhfr*, *pvdhps*, *pvmdr1* and *pvcrt*-*o* fragments analysed in the population from Colombia’s southern Amazon region had low genetic diversity; such polymorphisms seemed to be neutral according to Tajima, Fu and Li and d_N_–d_S_ tests. However, the Fay and Wu H estimator gave significant *pvdhfr* and *pvmdr1* values, suggesting a selective sweep. A candidate-resistant gene (*pvdhps*) having this pattern has been recorded previously in Colombia’s Córdoba department [17] according to information regarding the complete genome of *P. vivax* clinical isolates. Some mutations found in these *P. vivax* genes could have been fixed by conferring an advantage, in turn fixing neutral variants and thus reducing genetic diversity. As Fay and Wu tests gave statistically significant values, the southern Colombian Amazonian region’s parasite population might be under selective pressure, probably exerted by anti-malarial drugs; a selective sweep might thereby be fixing mutations enabling the appearance of resistant phenotypes. However, genes involved in selective sweep were different between Tierra Alta and the Colombian Amazonian region, probably due to differential treatment in these populations or populations in Colombia being structured with low gene flow between them, as seen in previous studies [54–56]. Further analysis is required to assess the selective sweep hypothesis regarding these genes in the Colombian Amazonian region.

## Conclusions

Even though studies in malaria-endemic regions have focused on the search for alternatives for improving the available treatment against malaria, the impact of these strategies on the evolution of *P. vivax* populations must be evaluated, due to the appearance and dispersion of resistance in such regions. Although, resistance is not fully understood concerning *P. vivax* and resistance-markers have not been clearly defined yet [16], *dhfr*, *dhps*, *mdr1* and *crt*-*o* genes have mutations which (in some cases) seem to provide resistance or at least reduced susceptibility to anti-malarial drugs [57]. Even though not all four genes have confirmed roles regarding *P. vivax* drug susceptibility, they are potential targets for *P. vivax* resistance. These four genes have low genetic diversity in the Colombian Amazon region. However, some of the mutations found were associated with resistance, i.e. the F1070L mutation in PvMDR1, S117N and S58R/K in PvDHFR and A383G and A382C in PvDHPS, whilst other new variants were also found in these genes. *pvmdr1* and *pvdhfr* fragments’ diversity pattern could be the consequence of a selective sweep, indicating that these genes’ variants might be becoming fixed in the population due to an adaptive advantage; these mutations could endow the parasite population with lower sensitivity to anti-malarial drugs. However, additional sequence analysis and in vitro assays are needed to confirm whether these variants are associated with resistance and/or therapeutic failures. The results reported here could be taken as a base tool for advising on how malaria control measures are implemented in Colombia and avoid the dispersion of haplotypes associated with resistance in this region.

## Abbreviations

*Pvmdr1*: *P. vivax* multidrug resistance 1 gene
*Pvdhfr*: *P. vivax* dihydrofolate reductase
*Pvdhps*: *P. vivax* dihydropteroate synthase
*Pvcrt*-*o*: *P. vivax* chloroquine resistance transporter
CQ: chloroquine
PQ: primaquine
SP: sulfadoxine–pyrimethamine
